# The prognostic value of Ki67 in ovarian high-grade serous carcinoma: an 11-year cohort study of Chinese patients

**DOI:** 10.18632/oncotarget.14112

**Published:** 2016-12-23

**Authors:** Ming Chen, Shuzhong Yao, Qinghua Cao, Meng Xia, Junxiu Liu, Mian He

**Affiliations:** ^1^ Department of Obstetrics and Gynecology, The First Affiliated Hospital, Sun Yat-Sen University, Guangzhou, China; ^2^ Department of Pathology, The First Affiliated Hospital, Sun Yat-Sen University, Guangzhou, China

**Keywords:** high-grade serous ovarian cancer, Ki67, platinum-resistance, survival, prognostic biomarker

## Abstract

**Objective:**

This study sought to assess the prognostic role of Ki67 in primary ovarian high-grade serous carcinoma (HGSC) and to determine whether Ki67 expression can predict responsiveness to platinum and paclitaxel chemotherapy.

**Results:**

A total of 318 women were included in the analysis and the median follow-up time was 48 months (range, 3–150 months). Ki67 proliferation indices ranged from 3% to 95% with a median of 40%. Using 40% as the cut-off value for the Ki67 index, we classified 141 patients as having low Ki67 expression and 177 patients as having high Ki67 expression. Low Ki67 expression was a predictor of platinum resistance (hazard ratio (HR) 2.85, 95% CI 1.43–5.98, *P* < 0.001). In the Kaplan-Meier analysis, comparisons of patients with low versus high Ki67 expression demonstrated that low Ki67 expression was significantly associated with decreased progression-free survival (PFS) (22% vs. 34% for 5-year PFS, *P* < 0.001) and decreased overall survival (OS) (31% vs. 55%, *P* < 0.001). Multivariate analysis indicated that low Ki67 expression was associated with decreased PFS (HR 2.98, 95% CI 1.75–6.56, *P* < 0.001) and decreased OS (HR 1.74, 95% CI 1.38–5.01, *P* = 0.003).

**Materials and Methods:**

A retrospective study of patients with stage I-IV primary ovarian HGSC was conducted from January 1, 2002, to December 31, 2012. Ki67 levels were measured via immunohistochemistry (IHC) and analyzed with respect to clinicopathological factors, and a survival analysis was performed.

**Conclusions:**

HGSC appears to be a heterogeneous disease with different clinical outcomes. Low Ki67 expression (< 40%) in HGSC is significantly associated with platinum resistance and decreased survival.

## INTRODUCTION

Although ovarian cancer accounts for only approximately 3% of cancer cases among women, it is one of the most deadly types of cancer. In 2015, approximately 21,000 women were diagnosed with ovarian cancer, and more than 14,000 women died of this disease [1]. The most common form of epithelial ovarian cancer is high-grade serous cancer (HGSC), which accounts for 60–80% of cases of epithelial ovarian cancer and the majority of epithelial ovarian cancer-related deaths [2]. Cytoreductive surgery followed by combination chemotherapy with platinum and paclitaxel has been regarded as the standard treatment for ovarian HGSC. However, recent data revealed that HGSC exhibits marked chromosomal aberrations and heterogeneous molecular and cellular biological characteristics [3–6]. These findings clearly indicate that one treatment will not be effective for all HGSC patients and challenge the “one-size-fits-all” concept with respect to treating HGSC. Therefore, it is essential to identify predictors that can distinguish the subsets of HGSC patients with relatively good prognoses who could benefit from conventional adjuvant therapy from those with the greatest need for more aggressive treatment regimens and/or targeted therapy.

The cellular proliferation status could reflect the proliferative potential of a tumor, as well as the sensitivity to chemotherapy; therefore, it is a potential prognostic tool. Ki67 is a nuclear located protein that is closely linked to cell proliferation. It is present during all active phases of the cell cycle but absent from resting cells [7]. Recently, Ki67 was identified as an important prognostic factor for many tumor entities with respect to chemosensitivity and disease recurrence/death [8–10]. For example, the St. Gallen Consensus Meetings have suggested the use of Ki67 expression as an index for classifying patients with breast cancer into different risk categories [11]. Moreover, carboplatin tends to be particularly effective in patients with certain subtypes of breast cancer, such as triple-negative breast cancer [12]. However, data regarding Ki67 in ovarian cancer are limited, and the prognostic value of Ki67 in HGSC remains controversial. Some investigations have considered higher Ki67 expression a risk factor for survival since highly proliferative tumors are associated with a worse outcome. However, other studies have indicated that HGSC patients with higher Ki67 expression tended to experience longer progression-free survival (PFS) because highly proliferative tumors seemed to respond better to first line chemotherapy [13–17]. As a result, the clinical value of Ki67 in ovarian cancer should be further explored.

The aim of our study was to investigate the prognostic value of Ki67 in HGSC and to describe the relationship between Ki67 expression and response to adjuvant chemotherapy with platinum and paclitaxel.

## RESULTS

### Basic characteristics of patients

The study cohort consisted of 318 patients with primary HGSC who underwent surgery at our hospital. All the patients were Chinese. The median age of the included patients was 59 years old (range, 25–82 years). The majority of the patients had advanced-stage disease (FIGO stages III–IV, 77.1%). Optimal cytoreduction was achieved in 71.3% of the patients (227/318). Complete pelvic lymphadenectomy was performed for 144 patients, 110 (76.4%) of whom underwent concurrent para-aortic lymphadenectomy. The median numbers of pelvic and para-aortic lymph nodes were 17 and 4, respectively. Complete pelvic lymphadenectomy was not performed due to the presence of advanced-stage disease (stage III–IV) in 174 patients, 54 of whom underwent lymph node sampling. All patients received adjuvant chemotherapy after surgery. Ultimately, 227 patients (71.4%) experienced recurrence during follow-up.

### Clinicopathological features of the Ki67-low and Ki67-high subgroups

**Figure 1:**
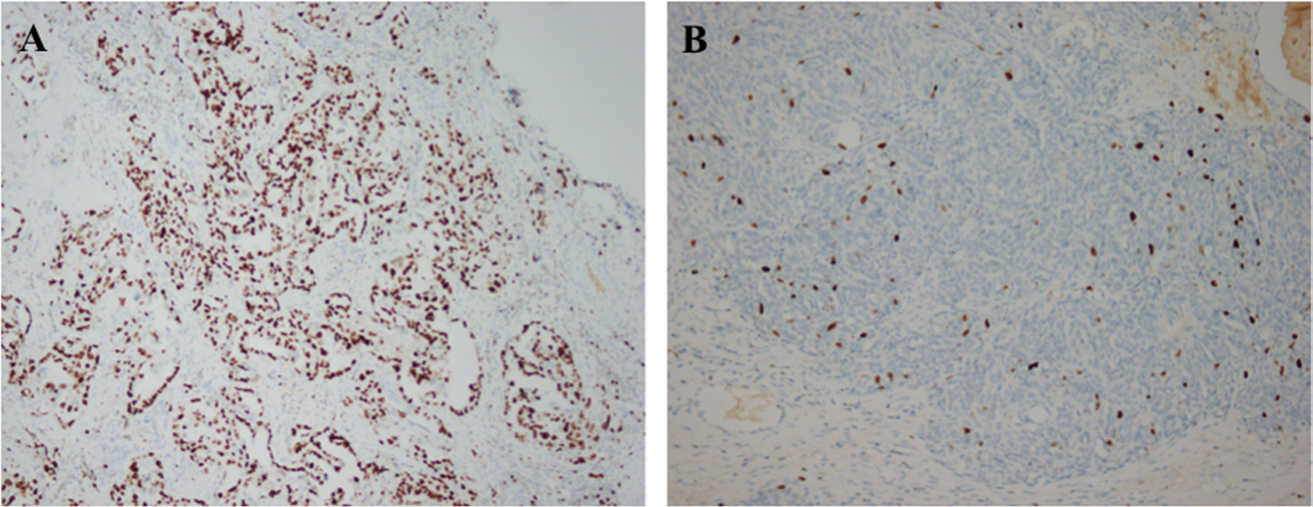
Immunohistochemical analysis of Ki67 expression in epithelial ovarian cancer (**A**) high expression, 100×; (**B**) low expression, 100×).

Ki67 proliferation indices ranged from 3% to 95% with a median of 40%. Using 40% as the cut-off value for the Ki67 index, we classified 141 patients (44.3%) as having low Ki67 expression and 177 patients (55.7%) as having high Ki67 expression (Figure 1). Patient characteristics for the subgroups stratified by Ki67 expression are presented in Table 1. In patients with HGSC, low Ki67 expression was significantly associated with the absence of lymphovascular space invasion (LVSI) (*P* = 0.004), a worse response to platinum-based chemotherapy (*P* < 0.001) and more recurrences (*P* = 0.005).

**Table 1:** Correlation of Ki-67 expression and patient characteristics

Variables	Total	Low Ki-67	High Ki-67	*P*
		(*N* = 141)	(*N* = 177)	
	(*n*, %)	(*n*, %)	(*n*, %)	
Age (y)				0.637
< 50	115 (36.2%)	53 (46.1%)	62 (53.9%)	
≥ 50	203 (63.8%)	88 (43.3%)	115 (56.7%)	
CA125				0.787
< 35	17 (5.3%)	7 (41.2%)	10 (58.8%)	
≥ 35	301 (94.7%)	134 (44.5%)	167 (55.5%)	
Menopausal status				0.874
Premenopausal	127 (39.9%)	57 (44.9%)	70 (55.1%)	
Postmenopausal	191 (60.1%)	84 (44.0%)	107 (56.0%)	
Performance status (ECOG)				0.451
0	182 (57.2%)	84 (46.2%)	98 (53.8%)	
≥ 1	136 (42.8%)	57 (41.9%)	79 (58.1%)	
Stage				0.642
I	25 (7.9%)	9 (36.0%)	16 (64.0%)	
II	48 (15.1%)	22 (45.8%)	26 (54.2%)	
III	225 (70.8%)	99 (44.0%)	126 (56.0%)	
IV	20 (6.3%)	11 (55.0%)	9 (45.0%)	
Lymph node metastasis (*N* = 198)				0.376
Negative	93 (29.2%)	43 (46.2%)	50 (53.8%)	
Positive	105 (33.0%)	42 (40.0%)	63 (60.0%)	
LVSI				**0.004**
Negative	121 (38.1%)	66 (54.5%)	55 (45.5%)	
Positive	197 (61.9%)	75 (38.1%)	122 (61.9%)	
Response to adjuvant chemotherapy				**< 0.001**
Platinum resistant	82 (25.8%)	71 (86.6%)	11 (13.4%)	
Platinum sensitive	236 (74.2%)	70 (29.7%)	166 (70.3%)	
Recurrence				**0.005**
Yes	227 (71.4%)	112 (49.3%)	115 (50.7%)	
No	91 (28.6%)	29 (31.9%)	62 (68.1%)	

Bold values indicate statistically significant differences.

LVSI: lymphovascular space invasion.

### Association between Ki67 expression and clinical outcomes

**Figure 2:**
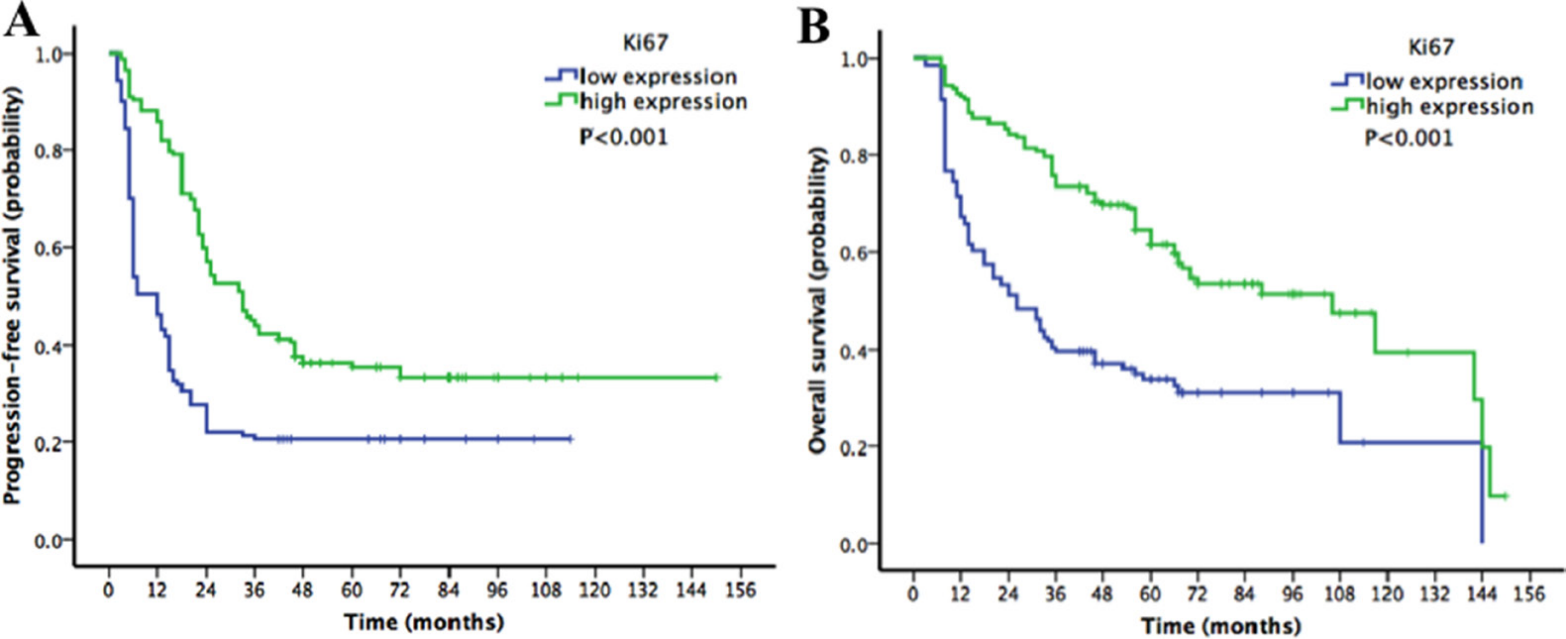
Kaplan-Meier survival curves for patients with epithelial ovarian cancer stratified by Ki67 expression (**A**) progression-free survival (PFS) curve; (**B**) overall survival (OS) curve). Low Ki67 expression was significantly associated with decreased PFS and decreased OS.

Survival analyses of patients in the Ki67-low and Ki67-high subgroups were performed. The 5-year PFS and OS rate for the whole cohort were 28% and 44%, respectively. In the Kaplan-Meier analysis, comparisons of patients with low versus high Ki67 expression demonstrated that low Ki67 expression was significantly associated with decreased PFS (20% vs. 35% for 5-year PFS, *P* < 0.001; Figure 2A) and decreased OS (31% vs. 55%, *P* < 0.001; Figure 2B).

Multivariate analysis was performed to identify relevant prognostic factors for HGSC patients (Table 2). Low Ki67 expression was associated with decreased PFS (HR 2.98, 95% CI 1.75–6.56, *P* < 0.001) and decreased OS (HR 1.74, 95% CI 1.38–5.01, *P* = 0.003). Moreover, Cox regression analysis revealed that advanced stage and suboptimal debulking surgery were associated with poor PFS and OS (advanced stage: PFS: HR 3.94, *P* < 0.001; OS: HR 4.13, *P* < 0.001; suboptimal debulking surgery: PFS: HR 2.37, *P* = 0.006; OS: HR 1.94, *P* = 0.02). LVSI was significantly related to worse PFS but not worse OS (PFS: *P* = 0.050; OS: *P* = 0.326).

**Table 2:** Multivariate analyses predicting survival in the cohort (*N* = 318)

Risk factor	PFS		OS	
	HR (95% CI)	*P*	HR (95% CI)	*P*
Age (y)		0.367		0.410
≥ 50	1		1	
< 50	0.78 (0.43–1.42)		0.84 (0.68–1.76)	
Stage		**< 0.001**		**< 0.001**
I–II	1		1	
III–IV	3.94 (1.74–8.67)		4.13 (1.92–9.23)	
Debulking		**0.006**		**0.02**
optimal	1		1	
suboptimal	2.37 (1.53–6.82)		1.94 (1.26–5.44)	
LVSI		**0.050**		0.326
Negative	1		1	
Positive	1.67 (0.998–4.30)		1.39 (0.67–3.79)	
Ki-67 expression		**< 0.001**		**0.003**
High	1		1	
Low	2.98 (1.75–6.56)		1.74 (1.38–5.01)	

Bold values indicate statistically significant differences.

PFS: progression-free survival; OS: overall survival; LVSI: lymphovascular space invasion; HR: hazard ratio; CI: confidence interval.

### Association of Ki67 expression with platinum resistance

**Figure 3:**
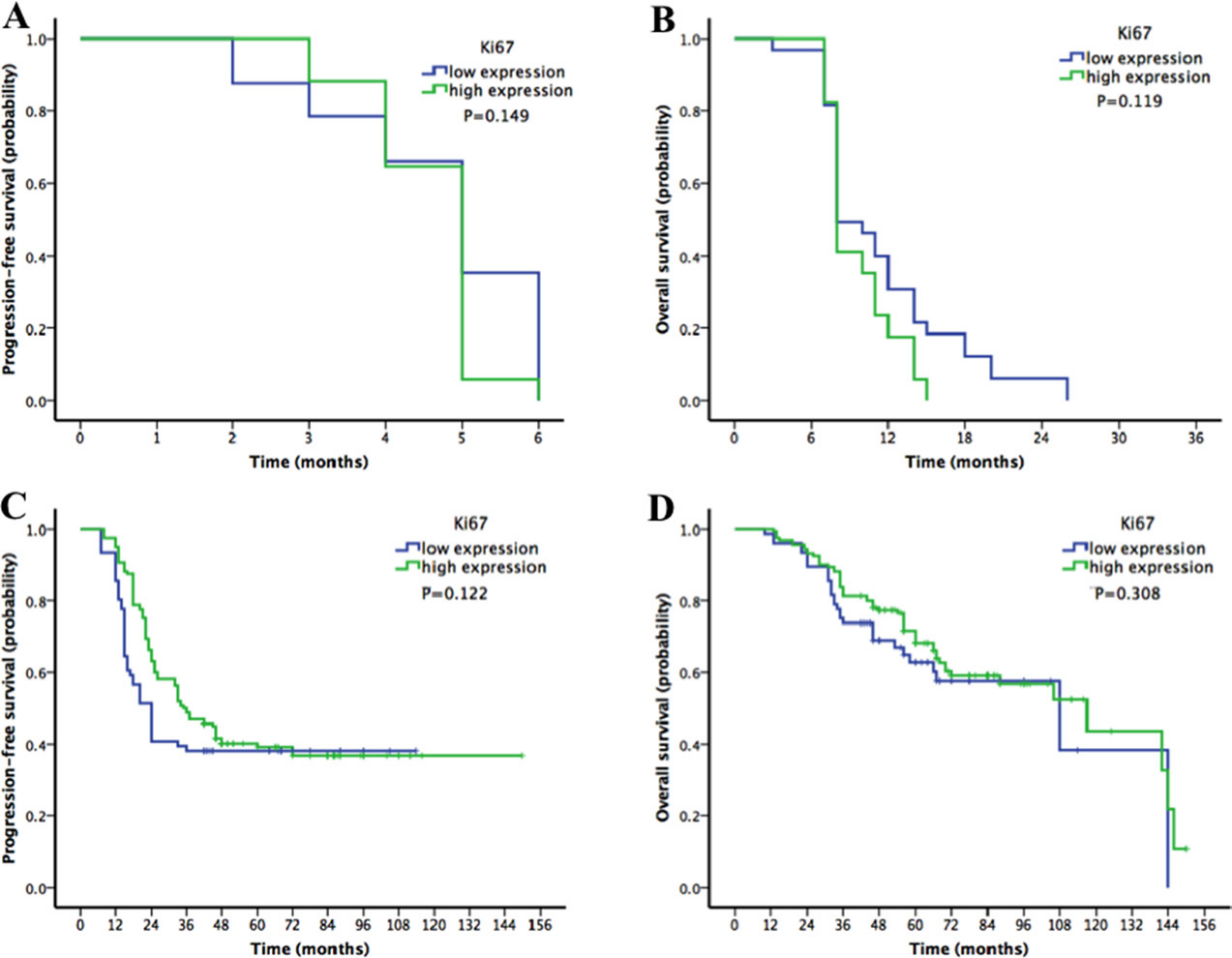
Kaplan-Meier survival curves for patients with epithelial ovarian cancer stratified by Ki67 expression in platinum-resistant and platinum-sensitive subgroups (**A**–**B**) progression-free survival (PFS) and overall survival (OS) in the platinum-resistant group according to Ki67 expression. (**C**–**D**) PFS and OS in the platinum-sensitive group according to Ki67 expression). There was no significant difference in PFS or OS according to Ki67 expression in either the platinum-resistant or the platinum-sensitive group.

Since most patients (*n* = 71, 86.6%) in the platinum-resistant group had low Ki67 expression, further analyses were performed to confirm the relationship between Ki67 and platinum resistance. When the patients were divided into platinum-resistant and platinum-sensitive groups, there was no significant difference in PFS or OS according to Ki67 expression (platinum-resistant group: PFS *P* = 0.149, OS *P* = 0.119, Figure 3A–3B; platinum-sensitive group: PFS *P* = 0.122, OS *P* = 0.308, Figure 3C–3D).

Then, the potential risk factors associated with platinum resistance in HGSC were evaluated (Table 3). In the univariate analysis, age, extent of debulking surgery and Ki67 expression were relevant risk factors. Subsequently, the multivariate analysis demonstrated that suboptimal surgery (HR 2.62, 95% CI 2.14–5.43, *P* < 0.001) and low Ki67 expression (HR 2.85, 95% CI 1.43–5.98, *P* < 0.001) were significantly associated with platinum resistance.

**Table 3:** Risk factors for platinum resistance in high-grade serous ovarian carcinoma

Variables	Platinum resistance (*n*, %)	Univariate		Multivariate	
		HR (95% CI)	*P*	HR (95% CI)	*P*
Age (y)			**0.036**		0.24
< 50	38 (33.0%)	1		1	
≥ 50	44 (21.7%)	0.77 (0.34–0.86)		0.85 (0.42–1.78)	
Menopausal status			0.743		
Premenopausal	34 (23.6%)	1			
Postmenopausal	48 (25.1%)	0.81 (0.40–1.67)			
Performance status (ECOG)			0.308		
0	43 (23.6%)	1			
≥ 1	39 (28.7%)	1.48 (0.77–3.53)			
CA125			0.17		
< 35	2 (11.8%)	1			
≥ 35	80 (26.6%)	2.52 (0.83–4.19)			
Stage			0.35		
I–II	8 (11.0%)	1			
III–IV	74 (30.2%)	2.14 (0.68–3.27)			
Lymph node metastasis			0.12		
Negative	17 (18.3%)	1			
Positive	29 (27.6%)	1.67 (0.78–2.85)			
LVSI			0.13		
Negative	25 (20.7%)	1			
Positive	57 (28.9%)	1.62 (0.84–2.19)			
Debulking			< 0.001		< 0.001
Optimal	48 (21.1%)	1		1	
Suboptimal	34 (37.4%)	2.62 (2.14–5.43)		3.54 (2.06–6.27)	
Ki-67 expression			< 0.001		< 0.001
High	65 (46.1%)	1		1	
Low	17 (9.6%)	3.47 (1.76–6.51)		2.85 (1.43–5.98)	

Bold values indicate statistically significant differences.

LVSI: lymphovascular space invasion.

## DISCUSSION

HGSC accounts for the vast majority of cases of ovarian carcinomas. This type of tumor is aggressive and typically discovered at an advanced stage; thus, these patients are usually treated with chemotherapy [18]. The identification of prognostic factors would facilitate the selection of adjuvant therapies by clinicians. This study produced the following key findings in HGSC: (i) a cut-off of 40% for Ki67 positivity was found to be prognostically discriminative with respect to PFS and OS and (ii) low tumoral Ki67 expression (< 40%) was a risk factor for platinum resistance.

The use of Ki67 as a prognostic marker has been widely investigated in the context of breast cancer but rarely addressed in the context of ovarian cancer. It was postulated that Ki67 levels would be increased in the entity of HGSC because much greater mitotic activity is observed in HGSC than in low-grade serous carcinoma (LGSC), and a mitotic index greater than 12/10 high-power microscopic fields (HPFs) favors a diagnosis of HGSC [19]. Kuhn et al. reported that the median Ki67 index in HGSCs was 37.6% [20], whereas Liu P et al. [14] found that 77.7% of epithelial ovarian cancer samples exhibited an immunoreactivity of greater than 50% [14]. In our study, the median Ki67 expression level was 40%, a finding consistent with the results of prior studies. However, the Ki67 level was not correlated with advanced disease in HGSC, as we observed no relationship between Ki67 expression and either FIGO stage or lymph node metastasis; previously, Kuhn et al detected no progressive increases in the Ki67 index in serous tubal intraepithelial carcinoma (STIC) and HGSC. These findings suggest that more rapid proliferation is not necessarily a feature of tumor progression in HGSC [20].

However, low Ki67 expression was associated with chemotherapy resistance and disease recurrence in our study. of high-grade tumorstheir Platinum-based chemotherapy is currently the mainstay of therapy for HGSC. Many clinical trials, including the ICON1 and ACTION trials demonstrated that the survival of ovarian cancer patients improves when adjuvant chemotherapy is added to the surgical procedure; thus, chemotherapy resistance would undoubtedly lead to shorter survival times [21, 22]. Although HGSC is more sensitive to chemotherapy, compared to other histotypes such as clear cell and mucinous carcinoma, appoximately 25% of patients experience primary platinum-resistance [23, 24]. In our cohort, the platinum-resistance rate was 25.8%, and low Ki67 expression was more common in platinum-resistant patients (86.1%). Platinum compounds cause DNA crosslinking and trigger apoptosis in tumor cells; in contrast, paclitaxel causes the formation of unusually stable microtubules and triggers the mitotic spindle checkpoint, resulting in apoptosis [25, 26]. These two cytotoxic drugs exhibit tumor-specific effects by preferentially killing highly proliferative cells. Slowly growing cells might survive platinum-paclitaxel chemotherapy and contribute to a negative outcome. Consistent with our result, a study by Feng et al. [13] involving 875 consecutive HGSC patients found that women with Ki67 indices greater than 50% had a longer PFS than women with Ki67 indices less than 50% (*P* = 0.021). In addition, Bachmayr-Heyda et al. [27] reported that the risk of mortality was more than three times higher among epithelial ovarian cancer patients with no Ki67^+^ cells than among those with Ki67^+^ cells (HR 3.34, 95% CI 1.59–7.04). Thus, if women with low Ki67 index HGSC were identified at the time of primary surgery, these patients could be treated with alternative drugs, such as angiogenesis inhibitors, which reduce tumor growth by inhibiting blood vessel formation rather than targeting rapidly proliferating cells [28].

Ki-67 measurement by IHC is a low-cost approach suitable for widespread use in clinical practice. In breast cancer, a tumor with a Ki67 index > 14–20% is regarded as highly proliferative [11, 29]. However, relative to breast cancer, epithelial cancers, particularly HGSC, tend to exhibit more aggressive biological behavior; therefore, a higher cut-off value should be used for these types of cancer. Since the Ki-67 index forms a continuous distribution, a clear definition of a single useful cut-off point is difficult. We selected the median Ki67 index (40%) as the cut-off value in this study, in accordance with the recommendations of the 2015 St. Gallen Conference [30]. Therefore the cut-off value of Ki67 expression level was 40% in our study. In the subsequent survival analyses, there was no significant differences in PFS or OS according to Ki67 expression when the patients were divided into platinum-resistant and platinum-sensitive groups. A Ki67 expression cut-off value of 40% helped identify those that might be sensitive to chemotherapy, but the chemotherapeutic response did not increase continually as the Ki67 index increased. Given inter-observer and inter-laboratory variability as well as the complexity of chemotherapy responses, the Ki67 cut-off value for HGSC requires additional exploration.

Our data fit the proposed heterogeneous model for HGSC and reveal a potential prognostic factor for chemotherapeutic response and survival. One strength of this study is that it is one of the first investigations to examine the relationship between Ki67 expression and chemotherapy responsiveness in HGSC. Moreover, the use of IHC rather than tissue microarrays (TMAs) for evaluating Ki67 expression is more suitable for clinical practice. The main limitations of this study are its retrospective nature and single-center design.

In conclusion, HGSC appears to be a heterogeneous disease with different clinical outcomes. In HGSC, low Ki67 expression (< 40%) is significantly associated with platinum resistance and decreased survival. The prognostic value of Ki67 expression in HGSC merits further exploration.

## MATERIALS AND METHODS

### Study group

We retrospectively reviewed the records from the First Affiliated Hospital of Sun Yat-sen University, China, to gather data pertaining to patients with primary ovarian HGSC who were treated between January 1, 2002, and December 31, 2012. All patients with stage I or II tumors underwent complete surgical staging. Optimal cytoreduction was performed for women with advanced (stage III or IV) cancer, with the exception of patients with unresectable tumors; these patients underwent suboptimal operations, and residual macroscopic tumors with maximal diameters greater than 1.0 cm remained. Staging was based on final pathological findings and determined according to the 2014 Federation of Obstetrics and Gynecology (FIGO) classification system [31]. Clinical and pathological variables included patient age, surgical procedure, and final pathology results (histological type and grade). Written informed consent was obtained from all participants. Study approval was obtained from the medical ethics committees at the First Affiliated Hospital of Sun Yat-sen University.

Patients who received no follow-up after surgery and patients who did not receive complete adjuvant chemotherapy as indicated or recommended were excluded from this study, as were patients who died within 30 days of surgery due to severe operative complications.

Pathology slides for all cases were subjected to hematoxylin and eosin (H&E) staining and were re-reviewed by two gynecologic pathologists. Diagnoses of HGSC were based on the MD Anderson Cancer Center (MDACC) two-tier grading system proposed by Malpica et al. [32]. The pathologists who evaluated the slides from the examined cohort were completely blinded to patient clinical information.

### Immunohistochemistry (IHC)

IHC was performed on 4 μm formalin-fixed, paraffin-embedded tissue sections of surgical specimens using a primary antibody against Ki67 (mouse monoclonal antibody, clone MIB-1, dilution 1:100; Dako). Tumor cell staining alone was evaluated; microenvironment staining was ignored. Ki67 proliferation indices were manually scored by two pathologists who determined overall percentages of Ki67^+^ cells in epithelial tumor tissue. Staining intensity was not relevant for this study [33].

### Treatment and follow-up

After surgery, all patients received adjuvant chemotherapy with platinum and taxane in accordance with the 2015 National Comprehensive Cancer Network (NCCN) ovarian cancer guidelines [34]. The carboplatin dose was calculated based on a target area under the curve of 5, and cisplatin was administered at a dose of 75 mg/m^2^. Paclitaxel was administered at a dose of 135–175 mg/m^2^. Patients who relapsed within 6 months of completing first-line therapy were classified as “platinum resistant”, as described in a prior study [35].

The median follow-up time was 48 months (range, 3–150 months). The patients were examined every 3 months for the first 2 years, every 6 months for the next 3 years, and yearly thereafter. Dates of recurrence were determined based on clinical examinations, imaging studies, and CA 125 levels. PFS was defined as the time interval from the date of primary surgery to the date of first disease recurrence. Overall survival (OS) was defined as the number of months from the date of primary surgery to the date of death. Survival was censored at the closeout date (May 1, 2016).

### Statistical analyses

Associations between variables and groups based on Ki67 expression were analyzed using chi-square tests. Risk factor associated with platinum resistance were assessed by the binary logistic regression test. Multivariate analysis with logistic regression was performed using the conditional forward method to identify the independent risk factors for platinum resistance. Survival curves were constructed using the Kaplan-Meier method. The prognostic values of the clinicopathological parameters with respect to PFS and OS were evaluated via the multivariate analysis (Cox proportional hazard regression test), with the conditional forward method used if applicable, and expressed as hazard ratios (HRs) and 95% confidence intervals (CIs). Statistical analyses were performed using SPSS 20.0 software (SPSS, Inc., Chicago, IL, USA). All tests were two-tailed and results with *P* <0.05 were regarded as statistically significant.
